# The Demographics of Non-motor Vehicle Associated Railway Injuries Seen at Trauma Centers in the United States 2007 - 2014

**DOI:** 10.7759/cureus.5974

**Published:** 2019-10-23

**Authors:** Christopher A Schneble, Jodi Raymond, Randall T Loder

**Affiliations:** 1 Orthopaedic Surgery, Yale University School of Medicine/Yale New Haven Hospital, New Haven, USA; 2 Pediatric Surgery, Riley Hospital for Children, Indianapolis, USA; 3 Orthopaedic Surgery, Riley Hospital for Children, Indianapolis, USA

**Keywords:** railway, injury, demographics, fatality, alcohol, drugs, injury severity score (iss)

## Abstract

Introduction

The majority of railway injury studies are limited by small sample size, restricted to a small geographical distribution, or located outside the United States (US). The aim of our study was to assess the demographic patterns associated with non-motor vehicle railway injuries in the US using a national trauma center database.

Materials and Methods

Data from the National Trauma Data Bank data from 2007 - 2014 were used; 3,506 patients were identified. For all statistical analyses, a p-value < 0.05 was considered significant.

Results

The patients were 81% male with an average age of 38.6 + 17.1 years and an Injury Severity Score (ISS) of 16.8 + 13.8. Males compared to females were younger (37.7 vs 42.5 years, p = 0.000002), had greater length of stays (12.7 vs 9.8 days, p = 0.000006), and higher ISS scores (17.1 vs 15.4, p = 0.0007). The geographic distribution within the US was most common in the South (32.0%) and least in the Northeast (18.9%). The racial composition was 67.5% White, 19.1% Black, 11.5% Hispanic/Latino, and 1.9% others. The most common mechanisms of injury were hitting/colliding with rolling stock (38.6%), followed by a fall in or from a train (19.5%), and collision with an object (13.5%). The majority of patients were pedestrians or passengers (68.5%); employees accounted for 12.5%. Although the majority were pedestrian/passengers for all regions, the Midwest had a greater proportion of employees (22.0%) compared to the other regions (7.8% to 12.2%) (p < 10^-6^), and thus injuries were more commonly work-related (24.6% vs 6.7% - 13.7%, p < 10^-6^). Work-related injuries were less severe (ISS 11.2 vs 17.3 - p < 10^-6^) and more commonly occurred due to a fall (32.8% vs 17.9%, p < 10^-6^). Alcohol and/or drug involvement was present in 40.7% and was less in those with work-related injuries (2.2%). Overall mortality was 6.4% and was less in those having a work-related injury (2.0 vs 6.6% p = 0.000004).

Conclusion

For non-motor vehicle USA railway injuries, the average age was 38.5 years; 80.6% were male. The injuries were least common in the Northeast and most common in the South. Racial distribution mirrored that of the US population. Alcohol involvement was present in 29%, lower than in previous studies. Mortality was 6.4%, also lower than previously reported.

## Introduction

Trains are a common form of transportation in the modern world for both passenger and freight carriage. Regarding passenger traffic, the Worldwide Railway Organization reported that in 2016, 7.342 billion passengers were carried on trains in Europe [1]. Europe accounts for 16% of worldwide passenger travel, giving a worldwide number of 45.9 billion passengers (45.9 billion = 7.342 billion/0.16). According to the 2016 United States (US) Bureau of Transportation Statistics, there were 39.61 billion passenger-miles for all rail modalities (Amtrak, commuter rail, and transit rail) [2]. The numbers for freight were somewhat different. In 2016, European railways carried 2.209 billion tons of freight [1], or 6% of worldwide freight, giving a worldwide freight carriage of ~36.8 billion tons (36.8 billion = 2.209 billion/0.06). Such massive volumes of transportation expose humans to significant potential for injury. When such injuries occur, they are associated with significant morbidity and mortality. In the last 10 years within the US, 119,303 railway injury events occurred, resulting in 87,106 nonfatal injuries and 7,451 deaths [3]. 

The majority of studies involving railway injuries are limited by small sample size, restricted to a small geographical distribution, and located outside the US [4-7]. Sousa et al. [7], Moore et al. [5], and Cina et al. [8] focused only on train-pedestrian events, while Shapiro et al. [4] and Hedelin et al. [6] reported on multiple mechanisms of injury. Maclean et al. [9] and Mohanty et al. [10] focused on only traumatic amputation patients and patients who perished, respectively. The aim of this investigation was to study the demographic patterns associated with non-motor vehicle collision railway injuries seen at trauma centers in the entire US using a national database. As this was an exploratory demographic study, there were no null hypotheses.

## Materials and methods

**Figure 1:**
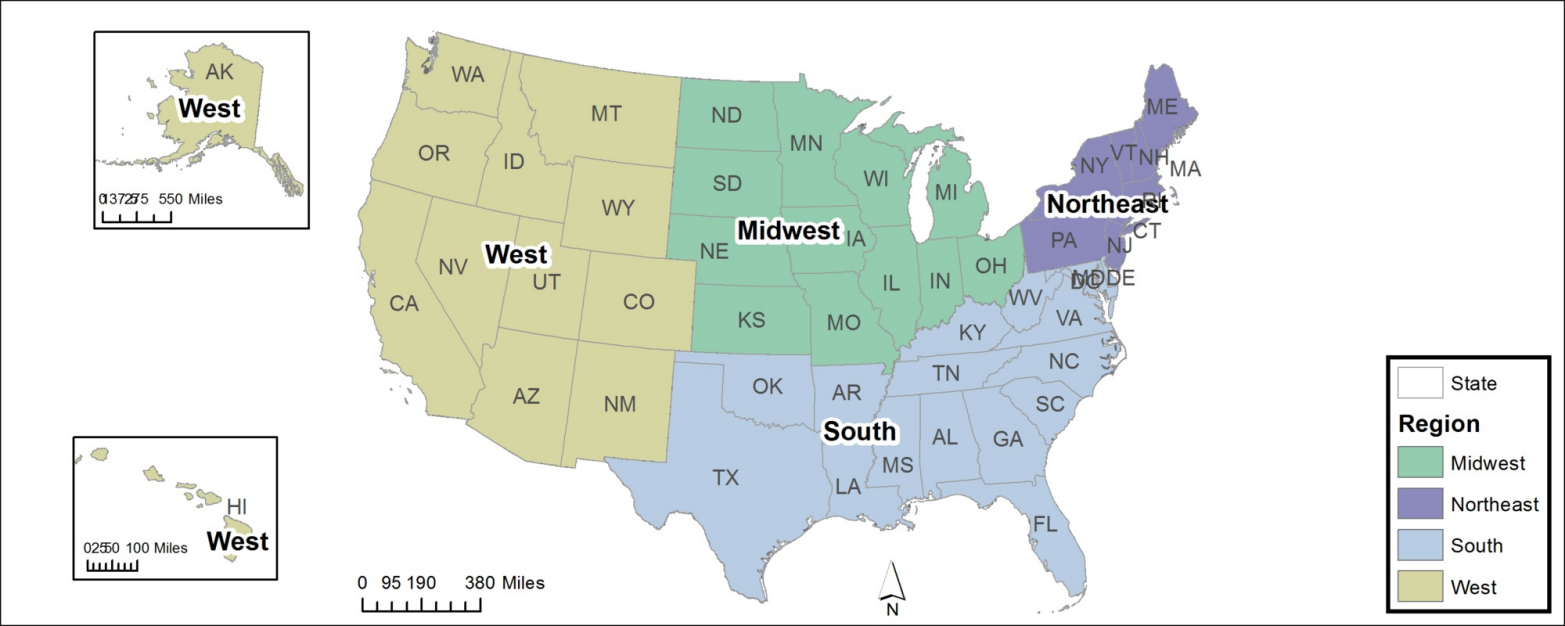
The four geographic regions as determined by the United States Census Bureau (http://www.census.gov/geo/reference/webatlas/regions.html) Figure obtained from the public domain, courtesy of the United States Census Bureau

The data for this study were obtained from the National Trauma Data Bank (NTDB) for the time period 2007 - 2014 and extracted using SAS version 9.4 (SAS Institute, Cary, NC) and further refined with Excel® 2013 (Microsoft®, Redmond, WA). There were 3,506 patients identified using the International Classification of Disease (ICD), 9th edition E codes for railway injuries (E800 - E807). These codes exclude those involving motor vehicles. Due to the nature of the NTDB, all patients presented to a hospital. The data collected were age, gender, ethnicity, region/location, length of stay (LOS), length of Intensive Care Unit (ICU) stay, ICD-9 diagnosis codes, ICD-9 procedure codes, complications, relation to work, association with alcohol or drug use, Injury Severity Score (ISS), and disposition from the hospital. Regions within the US were categorized as Northeast, West, South, and Midwest as determined by the US Census Bureau (Figure 1). This study was deemed exempt by the Indiana University Institutional Review Board (IRB00000220|IRB-01|Study #1705380487).

Continuous data are reported as the mean ± one standard deviation. Categorical data are reported as frequencies and percentages. Analyses between groups of continuous data were performed using non-parametric tests due to non-normal data distribution (Mann-Whitney U - two groups; Kruskal-Wallis test - three or more groups). Differences between groups of discrete data were analyzed by the Fisher’s exact test (2 x 2 tables) and the Pearson’s χ^2 ^test (greater than 2 x 2 tables). For all statistical analyses, p < 0.05 was considered statistically significant. Statistical analyses were performed with Systat 10™ (Systat Software, Inc., Chicago, IL).

## Results

Patient demographics

Most patients were male (80.5%) with an average age of 38.6 ± 17.1 years (range: 0 - 89 years) (Table 1). The mean length of stay (LOS) was 12.1 ± 17.7 days (range: 1 - 304 days). An ICU admission occurred in 1,563 (44.6%) of the patients with an average stay of 8.6 ± 11.1 days (range: 1 - 136 days). The average ISS was 16.8 ± 13.8; 43.3% were < 15 and 56.7% were > 16. Racial distribution (when known) was 67.5% White, 19.1% Black, 11.5% Hispanic/Latino, 1.8% Asian, and 0.1% Polynesian. The injuries occurred most commonly in the South (32.0%) and with the fewest in the Northeast (18.9%). The most common mechanisms of injury were hitting/colliding with rolling stock (38.6%), followed by a fall in or from a train (19.5%), and collision with an object (13.5%). The ICD9 defines ‘hit by rolling stock’ as being crushed by the train or any part of the train, injured or killed by the train, knocked down by the train, or run over by the train, excluding a pedestrian being hit or by objects set in motion by the train; ‘collision with rolling stock’ includes a collision between railway trains or railway vehicle, or any derailment of rolling stock colliding with other rolling stock. When known, the majority of patients were pedestrians or passengers (68.5%); employees accounted for 12.5%. The geographic location where the injury occurred was “other” (47.7%) (which likely means the railroad itself), followed by the street (22.7%), industry (11.1%), and seven other locations comprising the remainder. Alcohol and/or drug involvement was present in 1,428 patients (40.7%). Alcohol presence was confirmed in 1,001 (28.6%) and illicit drugs in 661 (18.9%); 264 (7.5%) had both alcohol and drug involvement. The injury was fatal in 223 (6.4%); 69 (2.0%) arrived with no signs of life, while 105 (3.0%) died in the emergency department (ED). The remaining 49 patients died at some point during their hospital stay prior to discharge. The payor was the government in 40.2%, self-pay in 22.8%, private/commercial insurance in 15.4%, and other payors for the remainder. Differences between various demographic groups are given below, understanding that with such a large data set some of the differences are statistically significant but may not be clinically significant.

**Table 1:** Overall Demographics by Gender and by Race * average for continuous variables; % for categorical variables ED: Emergency Department; ICU: Intensive Care Unit; ISS: Injury Severity Score; LOS: length of stay; SD: standard deviation; US: United States

Variable	All	Mean/%*	Male (M)	Female (F)	% M	% F	p-value	White (W)	Black (B)	Hispanic/Latino (H/L)	% W	%B	% H/L	p-value
All	-	-	2,522	610	80.5	19.5	-	2,115	599	359	68.8	19.5	11.7	-
Age (yrs ± 1 SD)	3,491	38.6 ± 17.2	37.7 ± 16.1	42.5 ± 20.7	-	-	0.000002	39.6 ± 17.4	37.4 ± 16.5	34.4 ± 15.3	-	-	-	< 10^-6^
LOS (days)	3,495	12.1 ± 17.6	12.7 ± 18.3	9.8 ± 14.0			0.000006	11.2 ± 16.1	13.8 ± 19.8	13.9 ± 19.3				0.052
ICU LOS (days)	1,563	8.6 ± 11.1	8.8 ± 11.6	7.6 ± 8.6			0.27	8.3 ± 10.2	10.1 ± 13.2	7.8 ± 12.6				0.004
ISS	3,410	16.8 ± 13.8	17.1 ± 13.9	15.4 ± 12.3			0.0007	16.6 ± 13.4	17.0 ± 14.8	17.0 ± 14.0				0.69
ISS Group														
< 15	1,449	43.3	1,199	250	44.2	39.2	0.021	894	233	152	43.4	40.8	42.8	0.53
> 16	1,899	56.7	1,511	388	55.8	60.8		1,164	338	203	56.6	59.2	57.2	
Gender														
Male	2,522	80.5	-	-	-	-	-	1,689	488	309	79.9	81.5	86.1	0.020
Female	610	19.5	-	-	-	-	-	426	111	50	20.1	18.5	13.9	
Race														
White	2,115	67.5	1,689	426	67.0	69.8	0.0001	-	-	-	-	-	-	
Black	599	19.1	488	111	19.3	18.2		-	-	-	-	-	-	
Hispanic/Latino	359	11.5	309	50	12.3	8.2		-	-	-	-	-	-	
Asian	55	1.8	33	22	1.3	3.6		-	-	-	-	-	-	
Polynesian	4	0.1	3	1	0.1	0.2		-	-	-	-	-	-	
Location in US														
Midwest	643	20.5	542	94	21.4	16.0	0.00002	465	77	37	23.8	14.8	11.0	< 10^-6^
Northeast	595	18.9	456	136	18.0	23.1		379	83	42	19.4	15.9	12.5	
South	1,004	32.0	839	161	33.1	27.3		552	298	109	28.2	57.1	32.3	
West	896	28.6	698	198	27.5	33.6		558	64	149	28.6	12.3	44.2	
Railway Event														
Collision with stock	339	9.8	266	71	9.6	10.7	0.0015	214	54	29	10.1	9.0	8.1	0.001
Collision with object	466	13.5	375	89	13.5	13.4		287	103	36	13.6	17.2	10.0	
Derailment	65	1.9	45	20	1.6	3.0		38	8	6	1.8	1.3	1.7	
Fire/explosion	14	0.4	12	2	0.4	0.3		12	1	1	0.6	0.2	0.3	
Fall	675	19.5	524	148	18.8	22.4		455	89	65	21.5	14.9	18.1	
Hit by stock	996	28.8	844	147	30.4	22.2		573	193	111	27.1	32.2	30.9	
Other railway accident	667	19.3	528	138	19.0	20.8		403	105	78	19.1	17.5	21.7	
Unspecified	234	6.8	186	47	6.7	7.1		133	46	33	6.3	7.7	9.2	
Person														
Employee	375	12.5	344	30	13.8	6.0	0.00002	281	44	25	15.6	8.0	7.6	0.000001
Pedestrian/Passenger	2,061	68.5	1,690	360	67.6	71.4		1,168	412	231	64.7	74.6	70.4	
Cyclist	102	3.4	78	35	3.1	6.9		67	18	8	3.7	3.3	2.4	
Unknown	469	15.6	389	79	15.6	15.7		288	78	64	16.0	14.1	19.5	
Work-related														
No	2,714	87.1	2,110	604	84.4	98.2	< 10^-6^	1,621	471	300	84.7	89.5	91.7	0.0002
Yes	402	12.9	391	11	15.6	1.8		293	55	27	15.3	10.5	8.3	
Injury Location														
Home	74	2.2	42	32	1.5	4.9	< 10^-6^	59	6	4	2.8	1.0	1.1	0.00003
Farm	7	0.2	6	1	0.2	0.2		4	1	1	0.2	0.2	0.3	
Mine	17	0.5	17	0	0.6	0.0		13	4	0	0.6	0.7	0.0	
Industrial	377	11.1	354	23	12.9	3.5		267	56	31	12.8	9.4	8.8	
Recreation	59	1.7	44	15	1.6	2.3		50	3	3	2.4	0.5	0.8	
Street	769	22.7	589	180	21.5	27.5		452	150	93	21.7	25.3	26.3	
Public Building	288	8.5	223	65	8.2	9.9		148	46	32	7.1	7.8	9.1	
Residential	2	0.1	2	0	0.1	0.0		2	0	0	0.1	0.0	0.0	
Other	1,616	47.7	1,308	308	47.8	47.1		988	299	155	47.4	50.4	43.9	
Unknown	180	5.3	150	30	5.5	4.6		100	28	34	4.8	4.7	9.6	
Alcohol Involvement														
No (by test)	977	28.4	785	192	28.2	29.0	< 10^-6^	604	178	100	28.6	27.9	28.6	0.032
Yes above legal limit	794	23.1	694	100	25.0	15.1		479	115	101	22.6	28.1	22.6	
Yes, trace	207	6.0	170	37	6.1	5.6		123	36	27	5.8	7.5	5.8	
Unknown	1,464	42.5	1,131	333	40.7	50.3		909	270	131	43.0	36.5	43.0	
Drug Involvement														
No by test	572	16.6	471	101	16.9	15.3	0.071	332	89	68	15.7	14.9	18.9	0.51
Yes	661	19.2	550	111	19.8	16.8		419	124	66	19.8	20.7	18.4	
Unknown	2,209	64.2	1,759	450	63.3	68.0		1,364	386	225	64.5	64.4	62.7	
Disposition														
Died	223	6.5	187	36	6.7	5.4	0.23	130	45	23	6.1	7.5	6.1	0.78
Discharged	2,779	80.7	2,248	531	80.9	80.2		1,724	476	291	81.5	79.5	81.5	
Released from ED/Unknown	440	12.8	345	95	12.4	14.4		261	78	45	12.3	13.0	12.3	
Payor														
Government	1,384	40.2	1,127	256	40.5	38.7	< 10^-6^	839	252	139	39.7	42.1	38.7	< 10^-6^
Blue Cross Blue Shield	136	4.0	97	39	3.5	5.9		103	14	7	4.9	2.3	1.9	
Private/Commercial	529	15.4	385	144	13.8	21.8		379	71	36	17.9	11.9	10.0	
Self	784	22.8	676	108	24.3	16.3		443	168	118	20.9	28.0	32.9	
Other	230	6.7	174	56	6.3	8.5		117	43	24	5.5	7.2	6.7	
Unknown	380	11.0	321	59	11.5	8.9		234	51	35	11.1	8.5	9.7	

Analyses by gender and race

Males compared to females were younger (37.7 vs 42.5 years), had a greater LOS (12.7 vs 9.8 days), and ISS scores (17.1 vs 15.4) (Table 1). Males had a larger proportion of Hispanic/Latinos, injuries occurring in the Midwest and South, injuries that were work-related, hit by the rail stock, and alcohol involvement. Males had a greater percentage of self-pay and a lower percentage of private/commercial insurance. Regarding race, Hispanic/Latinos were the youngest (34.4 +15.3 years) and Whites were the oldest (39.6 +17.4 years) (p < 10-6). The geographic location of the injury was mostly in the South for Blacks, South, and West for Hispanic/Latinos, and uniform across all four regions for Whites. The patient being an employee was higher in Whites and pedestrian/passengers in Blacks and Hispanic/Latinos. An industrial location was more common in Whites (12.8%) compared to Hispanic/Latinos (8.8%). Alcohol involvement was highest in Blacks and lowest in Hispanic/Latinos. Self-pay was most common in Hispanic/Latinos (32.9%) and lowest in Whites (20.9%), with a concomitant increase in private/commercial insurance in Whites (17.9%) compared to Hispanic/Latinos (10.0%). There was no difference in ISS, drug involvement, or hospital disposition by race. 

Analyses by region of country and relation with work

Although the majority were pedestrian/passengers for all regions, the Midwest had a greater proportion of employees (22.0%) compared to the other regions (7.8 to 12.2%), and thus injuries were more commonly work-related (24.6% vs 6.7 - 13.7%) and occurring in industrial locations (17.6% vs 6.0 - 11.9%) (Table 2). Those that were work-related had less severe injuries (ISS: 11.2 vs 17.3) and more commonly occurred due to a fall (32.8% vs 17.9%). Mortality was less in those having a work-related injury (2.0 vs 6.6%). Alcohol involvement was less in those with work-related injuries (2.2%). 

**Table 2:** Demographics by Geographic Region and Work Relationship ED: Emergency Department; ICU: Intensive Care Unit; ISS: Injury Severity Score; LOS: length of stay; NWR: not work-related; SD: standard deviation; WR: work-related

Variable	Midwest (MW)	Northeast (NE)	South (S)	West (W)	%MW	%NE	%S	%W	p-value	Not Work-related	Work-related	% NWR	%WR	p-value
Age (yrs + 1 SD)	37.6 ± 16.5	39.4 ± 18.5	37.5 ± 16.1	39.8 ± 17.2	-	-	-	-	0.027	38.4 ± 17.7	41.5 ± 12.6	-	-	0.000002
LOS (days)	11.1 ± 13.7	13.0 ± 18.8	13.0 ± 16.7	11.9 ± 18.7	-	-	-	-	0.002	12.1 ± 16.9	10.2 ± 20.8	-	-	0.11
ICU LOS (days)	8.2 ± 12.6	10.1 ± 13.5	9.3 ± 11.1	7.6 ± 8.9	-	-	-	-	0.005	8.6 ± 10.6	8.4 ± 17.5	-	-	0.0055
ISS	16.1 ± 13.1	17.3 ± 14.6	16.8 ± 13.3	18.0 ± 14.3	-	-	-	-	0.045	17.3 ± 13.9	11.2 ± 9.7	-	-	< 10^-6^
ISS Group														
< 15	265	256	419	418	41.5	43.5	44.4	47.1	0.16	1196	94	45.1	24.0	< 10^-6^
> 16	374	333	524	469	58.5	56.5	55.6	52.9		1453	298	54.9	76.0	
Railway Event														
Collision with Stock	58	59	77	110	9.0	9.9	7.7	12.3	< 10^-6^	275	36	10.1	9.0	< 10^-6^
Collision with Object	82	73	152	122	12.8	12.3	15.1	13.6		397	31	14.6	7.7	
Derailment	11	9	19	7	1.7	1.5	1.9	0.8		49	13	1.8	3.2	
Fire/explosion	6	4	2	1	0.9	0.7	0.2	0.1		4	8	0.1	2.0	
Fall	118	139	165	183	18.4	23.4	16.4	20.4		486	132	17.9	32.8	
Hit by Stock	198	166	326	225	30.8	27.9	32.5	25.1		779	98	28.7	24.4	
Other	125	117	199	176	19.4	19.7	19.8	19.6		526	73	19.4	18.2	
Unspecified	45	28	64	72	7.0	4.7	6.4	8.0		201	11	7.4	2.7	
Person														
Employee	134	37	113	57	22.0	7.8	12.2	7.9	< 10^-6^	68	277	2.9	71.8	< 10^-6^
Pedestrian/Passenger	353	343	691	526	57.9	72.4	74.3	72.8		1792	43	77.3	11.1	
Cyclist	20	5	21	44	3.3	1.1	2.3	6.1		90	0	3.9	0.0	
Unknown	103	89	105	96	16.9	18.8	11.3	13.3		367	66	15.8	17.1	
Work-related														
No	454	430	791	776	75.4	92.5	86.3	93.3	< 10^-6^					
Yes	148	35	126	56	24.6	7.5	13.7	6.7						
Injury Location														
Home	21	8	18	11	3.3	1.4	1.8	1.3	< 10^-6^	71	0	2.6	0.0	< 10^-6^
Farm	2	0	1	2	0.3	0.0	0.1	0.2		5	2	0.2	0.5	
Mine	2	4	10	0	0.3	0.7	1.0	0.0		2	13	0.1	3.3	
Industrial	112	35	118	80	17.6	6.0	11.9	9.2		100	249	3.7	62.9	
Recreation	12	5	20	14	1.9	0.9	2.0	1.6		51	1	1.9	0.3	
Street	125	46	236	300	19.7	7.8	23.7	34.6		691	17	25.8	4.3	
Public Building	27	142	30	44	4.3	24.2	3.0	5.1		265	6	9.9	1.5	
Residential	0	0	2	0	0.0	0.0	0.2	0.0		2	0	0.1	0.0	
Other	306	315	495	374	48.2	53.7	49.8	43.1		1,351	93	50.4	23.5	
Unknown	28	32	64	43	4.4	5.5	6.4	5.0		142	15	5.3	3.8	
Alcohol Involvement														
No (by test)	197	149	262	281	30.6	25.0	26.1	31.4	0.00002	743	168	27.3	41.8	< 10^-6^
Yes above legal limit	163	135	232	193	25.3	22.7	23.1	21.5		717	3	26.4	0.7	
Yes, trace	28	25	61	78	4.4	4.2	6.1	8.7		187	6	6.9	1.5	
Unknown	255	286	449	344	39.7	48.1	44.7	38.4		1070	225	39.4	56.0	
Drug Involvement														
No by test	102	98	162	169	15.9	16.5	16.1	18.9	0.00003	461	62	17.0	15.4	< 10^-6^
Yes	117	78	231	186	18.2	13.1	23.0	20.8		582	39	21.4	9.7	
Unknown	424	419	611	541	65.9	70.4	60.9	60.4		1674	301	61.6	74.9	
Disposition														
Died	32	46	65	64	5.0	7.7	6.5	7.1	0.13	180	8	6.6	2.0	0.000004
Discharged	531	468	834	713	82.6	78.7	83.1	79.6		2160	360	79.5	89.6	
Released from ED/Unknown	80	81	105	119	12.4	13.6	10.5	13.3		377	34	13.9	8.5	
Payor														
Government	251	244	386	367	39.0	41.0	38.4	41.0	< 10^-6^	989	286	36.4	71.1	< 10^-6^
Blue Cross/Blue Shield	17	27	45	42	2.6	4.5	4.5	4.7		116	9	4.3	2.2	
Private/Commercial	104	123	113	144	16.2	20.7	11.3	16.1		436	49	16.0	12.2	
Self	135	89	325	164	21.0	15.0	32.4	18.3		703	6	25.9	1.5	
Other	19	35	72	64	3.0	5.9	7.2	7.1		200	11	7.4	2.7	

Analyses by the mechanism of injury and local geographic location

Those who were injured in a fall were older (43.8 years) than the other major mechanisms of injury (collision with the stock, collision with an object, hit by the stock, or other) (36.6 - 39.6 years) (Table 3). The LOS was highest for those who were hit by the stock (13.8 days vs 8.3 - 13.1); however, when an ICU stay occurred, those who collided with the stock had the highest LOS in the ICU (10.4 days vs 7.0 - 9.1). Those who had a collision with an object or were hit by the stock had more severe injuries (ISS: 19.1 and 18.8 vs 11.5 - 17.5). Alcohol involvement was more common in those who were hit by the stock (52.8%) than other mechanisms of injury. Hospital mortality was highest in those who collided with an object (8.6%) or were hit by the stock (8.2%). 

**Table 3:** Demographics by Mechanism of Injury and Local Geographic Location CO: collision with object; CS: collision with stock; ICU: Intensive Care Unit; Ind: industrial; ISS: Injury Severity Score; LOS: length of stay: Oth: other; PB: public building; SD: standard deviation; Str: street

Variable	Collision with Stock	Collision with Object	Fall	Hit by Stock	Other	% CS	%CO	%Fall	%Hit	%Oth	p-value	Industrial	Street	Pub Build	Other	%Ind	%Str	%PB	%Oth	p-value
Age (yrs ± 1 SD)	39.6 ± 16.9	37.1 ± 16.4	43.8 ± 19.6	36.6 ± 15.5	38.0 ± 16.5						< 10^-6^	39.0 ± 14.0	39.4 ± 17.2	43.1 ± 18.2	37.6 ± 16.9					0.00001
LOS (days)	11.9 ± 16.7	13.1 ± 18.4	8.3 ± 12.3	13.8 ± 17.3	12.9 ± 18.9						< 10^-6^	11.0 ± 20.7	11.9 ± 17.7	12.3 ± 18.1	12.8 ± 16.8					0.003
ICU LOS (days)	10.4 ± 14.0	9.1 ± 11.2	7.0 ± 9.8	8.0 ± 9.8	8.5 ± 10.9						0.0007	7.6 ± 13.6	8.8 ± 10.9	8.5 ± 9.6	8.6 ± 10.6					0.59
ISS	16.6 ± 13.2	19.1 ± 13.8	11.5 ± 9.9	18.8 ± 13.9	17.5 ± 15.3						< 10^-6^	12.9 ± 11.4	18.6 ± 14.3	15.5 ± 13.0	18.0 ± 14.4					< 10^-6^
ISS Group																				
< 15	140	233	165	489	287	43.2	51.8	24.7	50.6	44.2	< 10^-6^	112	366	114	743	30.5	48.7	39.7	47.2	< 10^-6^
> 16	184	217	503	478	363	56.8	48.2	75.3	49.4	55.8		255	386	173	832	69.5	51.3	60.3	52.8	
Railway Accident																				
Collision with Stock	-	-	-	-	-	-	-	-	-	-		32	71	15	150	8.5	9.2	5.2	9.2	< 10^-6^
Collision with Object	-	-	-	-	-	-	-	-	-	-		22	156	34	216	5.8	20.3	11.8	13.3	
Derailment	-	-	-	-	-	-	-	-	-	-		6	15	3	21	1.6	2.0	1.0	1.3	
Fire/Explosion	-	-	-	-	-	-	-	-	-	-		4	2	1	2	1.1	0.3	0.3	0.1	
Fall	-	-	-	-	-	-	-	-	-	-		127	77	91	304	33.7	10.0	31.6	18.7	
Hit by Stock	-	-	-	-	-	-	-	-	-	-		104	206	84	513	27.6	26.8	29.2	31.6	
Other Railway Accident	-	-	-	-	-	-	-	-	-	-		68	167	47	316	18.0	21.7	16.3	19.5	
Unspecified	-	-	-	-	-	-	-	-	-	-		14	75	13	100	3.7	9.8	4.5	6.2	
Injury Location																				
Home	32	5	2	15	11	9.6	1.1	0.3	1.5	1.7	< 10^-6^	-	-	-	-	-	-	-	-	
Farm	1	1	0	1	4	0.3	0.2	0.0	0.1	0.6		-	-	-	-	-	-	-	-	
Mine	2	4	3	0	6	0.6	0.9	0.5	0.0	0.9		-	-	-	-	-	-	-	-	
Industrial	32	22	127	104	68	9.6	4.8	19.3	10.6	10.3		-	-	-	-	-	-	-	-	
Recreation	8	3	10	10	5	2.4	0.7	1.5	1.0	0.8		-	-	-	-	-	-	-	-	
Street	71	156	77	206	167	21.3	33.9	11.7	21.0	25.4		-	-	-	-	-	-	-	-	
Public Building	15	34	91	84	47	4.5	7.4	13.8	8.6	7.1		-	-	-	-	-	-	-	-	
Residential	1	1	0	0	0	0.3	0.2	0.0	0.0	0.0		-	-	-	-	-	-	-	-	
Other	150	216	304	513	316	45.0	47.0	46.2	52.3	48.0		-	-	-	-	-	-	-	-	
Unknown	21	18	44	47	34	6.3	3.9	6.7	4.8	5.2		-	-	-	-	-	-	-	-	
Alcohol Involvement																				
No (by test)	83	149	200	271	183	24.5	24.4	20.2	21.4	19.8	< 10^-6^	143	229	66	447	37.9	22.0	29.8	27.6	< 10^-6^
Yes, above legal limit	63	103	105	318	142	18.6	16.9	10.6	25.1	15.4		28	198	81	421	7.4	11.9	25.7	26.0	
Yes, trace	18	175	342	338	299	5.3	28.6	34.6	26.7	32.4		11	51	21	100	2.9	5.1	6.6	6.2	
Unknown	175	184	342	338	299	51.6	30.1	34.6	26.7	32.4		195	291	120	654	51.7	61.0	37.8	40.3	
Drug Involvement																				
No by test	44	86	90	191	116	13.0	18.5	13.3	19.2	20.1	0.00003	59	131	51	282	15.6	11.9	17.0	17.4	0.00002
Yes	61	110	108	208	117	18.0	23.6	16.0	20.9	20.3		41	162	41	346	10.9	10.2	21.1	21.3	
Unknown	234	270	477	597	343	69.0	57.9	70.7	59.9	59.5		277	476	196	994	73.5	78.0	61.9	61.3	
Disposition																				
Died	15	40	25	82	37	4.4	8.6	3.7	8.2	5.5	0.0032	17	62	21	110	4.5	8.1	7.3	6.8	0.0005
Discharged	275	363	568	795	538	81.1	77.9	84.1	79.8	80.7		330	583	237	1301	87.5	75.8	82.3	80.2	
Released from ED/Unknown	49	63	82	119	92	14.5	13.5	12.1	11.9	13.8		30	124	30	211	8.0	16.1	10.4	13.0	
Payor																				
Government	141	161	300	411	259	41.6	34.5	44.4	41.3	38.8	0.0001	226	259	110	632	59.9	40.7	33.7	39.0	< 10^-6^
Blue Cross/Blue Shield	25	20	28	30	19	7.4	4.3	4.1	3.0	2.8		9	26	16	66	2.4	11.9	3.4	4.1	
Private/Commercial	61	91	100	135	101	18.0	19.5	14.8	13.6	15.1		58	105	63	237	15.4	18.6	13.7	14.6	
Self	61	123	127	248	157	18.0	26.4	18.8	24.9	23.5		37	204	48	420	9.8	13.6	26.5	25.9	
Other	18	32	39	50	58	5.3	6.9	5.8	5.0	8.7		8	79	22	105	2.1	5.1	10.3	6.5	
Unknown	33	39	81	122	73	9.7	8.4	12.0	12.2	10.9		39	96	29	162	10.3	10.2	12.5	10.0	

Mortality

The overall mortality was 6.4% (223 of 3,506). Significant differences between those who survived and those who died are shown in Table 4. Those who died had lower LOS and ICU LOS but a higher ISS. Those who were hit by the stock or collided with an object had higher mortality, as did pedestrian/passengers and cyclists, while employees and those with work-related injuries had a lower mortality rate. Those who were self-pay (likely uninsured) had a higher mortality rate, while those insured by government programs, Blue Cross/Blue Shield (BCBS), and private/commercial payors had a lower mortality rate.

**Table 4:** Demographics by Survival ^ only for those injuries occurring at industrial properties, street, public buildings, or other ICU: Intensive Care Unit; ISS: Injury Severity Score; LOS: length of stay; SD: standard deviation; US: United States

Variable	Survived	Died	% Survived	% Died	p-value
Age (yrs ± 1 SD)	38.6 ± 17.2	40.5 ± 17.5	-	-	0.13
LOS (days)	14.0 ± 17.7	4.7 ± 7	-	-	< 10^-6^
ICU LOS (days)	8.9 ± 11.5	5.7 ± 8.0	-	-	< 10^-6^
ISS	15.2 ± 11.5	38.2 ± 16.0	-	-	< 10^-6^
ISS Group					
< 15	1,631	13	60.0	6.1	< 10^-6^
> 16	1,088	199	40.0	93.9	
Gender	n	n			
Male	2,248	187	80.9	83.9	0.33
Female	531	36	19.1	16.1	
Race					
White	1,724	130	68.0	64.4	0.76
Black	476	45	18.8	22.3	
Hispanic/Latino	291	23	11.5	11.4	
Asian	43	4	1.7	2.0	
Polynesian	3	0	0.1	0.0	
Location in the US					
Midwest	531	32	20.9	15.5	0.18
Northeast	468	46	18.4	22.2	
South	834	65	32.8	31.4	
West	713	64	28.0	30.9	
Railway Accident					
Collision with Stock	275	15	9.9	6.7	0.00004
Collision with Object	363	40	13.1	17.9	
Derailment	60	0	2.2	0.0	
Fire/Explosion	8	0	0.3	0.0	
Fall	568	25	20.4	11.2	
Hit by Stock	795	82	28.6	36.8	
Other Railway Accident	538	37	19.4	16.6	
Unspecified	173	24	6.2	10.8	
Person					
Employee	332	6	13.0	2.9	< 10^-6^
Pedestrian/Passenger	1,607	174	63.1	84.1	
Cyclist	72	12	2.8	5.8	
Unknown	388	19	15.2	9.2	
Work-related					
No	2,160	180	85.7	95.7	0.00002
Yes	360	8	14.3	4.3	
Injury Location					
Home	66	1	2.4	0.5	0.052
Farm	7	0	0.3	0.0	0.073^
Mine	13	0	0.5	0.0	
Industrial	330	17	12.1	7.7	
Recreation	51	1	1.9	0.5	
Street	583	62	21.3	28.2	
Public Building	237	21	8.7	9.5	
Residential	2	0	0.1	0.0	
Other	1,301	110	47.5	50.0	
Unknown	147	8	5.4	3.6	
Alcohol Involvement					
No (by test)	807	55	29.0	24.7	0.42
Yes, above legal limit	672	56	24.2	25.1	
Yes, trace	159	17	5.7	7.6	
Unknown	1,142	95	41.1	42.6	
Drug Involvement					
No (by test)	492	34	17.7	15.2	0.024
Yes	576	32	20.7	14.3	
Unknown	1,712	157	61.6	70.4	
Payor					
Government	1,179	74	42.4	33.2	< 10^-6^
Blue Cross/Blue Shield	117	5	4.2	2.2	
Private/Commercial	450	24	16.2	10.8	
Self	565	81	20.3	36.3	
Other	181	5	6.5	2.2	
Unknown	288	34	10.4	15.2	

## Discussion

There is little literature studying non-motor vehicle railway injuries in the US; the available studies are narrow in scope with small sizes [5, 8, 10]. The previous non-motor vehicle associated railway injury studies [4, 6, 9-10] demonstrate a similar age: 39 years [6], 37 years [9], 31 years [4], and 21 - 40 years [10] compared to the 38.5 years in this study. There is also a male predominance in railway-associated injuries; we found that 80.6% of the injuries were in males, similar to 87% [4] and 59% [6] in other studies. Males appeared to have sustained more severe injuries as indicated by their increased ISS, LOS, and ICU days, despite a younger average age. The average hospital LOS in our study of 12 days is consistent with 11 days in Sweden [6].

This is the first study to examine racial, geographical, and billing differences within railway injuries. The racial distribution seen in this study resembles that of the general US population [11]. Although certain racial groups were more commonly injured in different regions, this likely represents the racial distribution within the US. Railway injuries were most common in the South and least in the Northeast, even though the Northeast has three of the top five busiest Amtrak stations accounting for 80% of the Amtrak passenger traffic (20,034,851 of 25,139,294 riders). The likely explanation for this is that the South has more miles of freight railroad compared to the Northeast, as the US is known to have much greater railway freight traffic compared to passenger traffic. In 2016, European railways carried 2.209 billion tons of freight, representing 6% of the worldwide rail freight; the worldwide percentage of freight carried for America was 29%, 36% for Asia, and 27% for Russia. By contrast, the Worldwide Railway Organisation reported that 7.342 billion passengers were carried on trains in Europe in 2016 [1], with the worldwide percentage of passenger travel being 16% for Europe, 78% for Asia/Oceania, and 1% for America. This poses the question as to whether most of these injuries in this study occurred on or around freight railways as compared to passenger trains. If that is so, then the 71.4% of the patients that were designated as pedestrians or passengers were likely pedestrians and not passengers on the train. They were also likely trespassers [12], although such data is not given in the NTDB.

Work-related injuries were more frequent in men and less severe than non-work-related injuries (ISS 11.2 vs 17.3), explaining the lower mortality rate (2.0% vs 6.6%). The discrepancy in ISS could also be related to a lower threshold of workers seeking medical evaluation and presenting with less severe injuries compared to those with non-work-related injuries. Work-related injuries also rarely involved alcohol and were more commonly the result of a fall when compared to non-work injuries (32.8% vs 17.9%). This increase in falls compared to non-work injuries could be related to employment needs/requirements, but the exact etiology is unclear. Interestingly, both work-related and non-work-related injuries had similar percentages for collision with stock (9.0% and 10.1%, respectively), and hit by stock (24.4% and 28.7%, respectively). This suggests that increased exposure and awareness of railways and their dangers do not necessarily decrease the incidence of stock-related injuries. 

The differences by injury mechanism deserve further discussion (Table 3). The least severe injuries were those due to a fall, and the more severe injuries were due to those patients that were hit/collided with the stock or collided with an object. Those sustaining a fall were the least likely to be injured on the street and had the highest proportion of being injured in a public building and industrial locations. Similarly, they were the oldest of the various groups when grouped by injury mechanism. This likely means that those injured in public buildings were riders on the train, and those injured in industrial locations employees with work-related injuries (Table 2). Conversely, those who were hit by stock more commonly sustained their injuries in “other” locations, which likely means the railroad itself. This may indicate that further passive governmental agencies (whether local, statewide, or national) create ways in which it is more difficult for non-sanctioned people to actually be on the railroad, such as more barriers to impede trespassers, etc. Active legislation will likely not work; passive measures are much better for injury prevention. 

The role of alcohol in railway injuries is variable in the literature. Hedelin et al. [6] noted that 21% of non-fatal and 60% of fatal injuries involved alcohol above the legal limit, while our findings noted an approximately equal percentage of both non-fatal and fatal injuries (24.2% and 25.1%, respectively). When recalculating the data of Hedelin et al. [6] using only the number of patients above the legal alcohol limit, the result is 23.9%, the same as our result of 24.2%. We noted that 29.1% of patients had some evidence of alcohol in their system compared to the 70% [4] and 80% [5, 8] in other studies. In India, alcohol was detected in only 2.8% of 88 railway-related death victims examined post-mortem [10]. All of the studies mentioned above have sample sizes lower than 250, which may explain these differences. The true percentage of patients under the influence of alcohol in train-related injuries is likely in the range corroborated by both our study and that of Hedelin et al. [6]. 

Previously published railway injury mortality rates are 17% [13], 14% [4], and 10.4% [6], compared to our 6.4%. One explanation for these differences is that this study included all injury mechanisms that were classified as involving a railway and its premises, except those involving motor vehicles. Therefore, this study was not limited to only train/pedestrian events as seen in two of the studies [6, 13]. Higher mortality rates would be expected in those studies, as they would involve more severe injury mechanisms (such as hit by rolling stock which resulted in a 36.8% mortality rate in this study) while excluding less severe ones. Shapiro et al. [4] included motor vehicles, making it difficult to appropriately compare mortality rates with other studies excluding those injuries. All of the studies above, as well as in the present study, use hospital records to identify the patient population, which, unfortunately, neglects those who died on the scene and never presented to a hospital [4, 6, 13]. Thus, it is likely that the true railway injury mortality rate is higher than reported in all of these studies. The exact magnitude of this difference is difficult to know.

Limitations

The first limitation of this study is that the data entered into the NTDB is only as accurate as those entering the data. A recent study noted that there is interhospital variability in data coding and scoring with an overall 64% accuracy [14]. The accuracy, however, was good for standard demographic variables (96%) and less accurate for physiologic variables, such as pre-hospital vital signs (36%). Thus, the data for this study is likely very accurate as this is primarily a demographic study. Second, details of the ICD-9E codes regarding the mechanism of injury is not given, e.g., where exactly was the patient when hit by the rolling stock, etc. The passenger/pedestrian groups are not further broken down in the ICD-9 coding, so we could not study these subgroups. Another limitation is that not all patients have data entered for every variable; this was especially true for associated alcohol and drug use. This study is biased towards the more severe injuries, as all these patients were seen at trauma centers. It is very likely the more minor injuries, such as simple fractures, were seen and cared for at non-trauma center hospitals. The magnitude of this is difficult to know. 

## Conclusions

In this large study of the demographics of non-motor vehicle collision railway injuries treated at trauma centers, the average age was 38.5 ± 17.1 years with a male predominance of 80.6%. The injuries were least common in the Northeast and most common in the Southern US. The racial distribution of the injured patients mirrored that of the US population. Alcohol involvement was present in 29.1% of patients, lower than in previous US studies. The overall mortality rate was 6.4%, also lower than previously reported. These findings can be used by all involved in the care of such patients, as well as those in injury prevention.
